# Assessment of Oxygen Expansion during Internal Bleaching with Enamel and Dentin: A Comparative In Vitro Study

**DOI:** 10.3390/dj9090098

**Published:** 2021-08-24

**Authors:** Alba Pallarés-Serrano, Sandra Pallarés-Serrano, Antonio Pallarés-Serrano, Antonio Pallarés-Sabater

**Affiliations:** Department of Endodontics and Restorative Dentistry, School of Medicine and Dentistry, Catholic University of Valencia, Quevedo 2, 46001 Valencia, Spain; sandrapallaresserrano@gmail.com (S.P.-S.); antoniopallaresserrano@gmail.com (A.P.-S.); antonio.pallares@ucv.es (A.P.-S.)

**Keywords:** hydrogen peroxide, tooth bleaching, tooth resorption, in-vitro

## Abstract

Internal bleaching is a conservative, non-invasive, and simple treatment that is frequently performed in daily clinical practice. The present in vitro study analyzes the oxygen expansion of different bleaching agents resulting from the oxidation reaction when interacting with enamel and dentin. Enamel and dentin were crushed separately until obtaining a fine powder with particles of an approximate size between 0.06 and 0.2 mm. Each enamel and dentin sample were mixed with 37% carbamide peroxide (CP 37%), 30% hydrogen peroxide (HP 30%), sodium perborate (SP) combined with HP 30% (HP 30% + SP) and SP with distilled water (SP). A total of 280 1 mm diameter glass tubes were used with 70 for each bleaching agent (30 for powdered enamel evaluation, 30 for powdered dentin evaluation, and 10 controls). The bleaching agents were placed in the prepared tubes immediately after mixing the components. As expansion occurred, the oil inside the tube was displaced, through which the resulting expansion was evaluated and measured for 10 days. A significant expansion was observed that varied in magnitude according to the bleaching agent and the tooth structure used. Student’s t test and Welch’s ANOVA were used to analyze the data obtained. The highest mean expansion of both enamel and dentin was observed with 30% HP (66.6 mm for enamel, 94.5 mm for dentin) followed by HP 30% + SP (48.6 mm for enamel, 52.7 mm for dentin), CP 37% (38.4 mm for enamel, 52.6 mm for dentin) and finally SP with water (12.7 mm for enamel, 4.4 mm for dentin). It was observed that the expansion in the SP group with enamel was significantly lower than in the rest of the groups, while that registered for HP 30% was significantly higher. (*p <* 0.001). The results with dentin were similar, with a significantly lower expansion for SP and higher for HP 30% (*p <* 0.001). The oxygen expansion observed as a result of the interaction between bleaching agents and dental tissues could contribute to improving our understanding of bleaching and its results. These results suggest that bleaching agents react with the organic component of the tooth structure.

## 1. Introduction

Assessment of dental color is one of the most critical components of cosmetic dentistry. [1] Darkening of a single anterior tooth produces a negative aesthetic effect due to the lack of coincidence in color with the rest of the teeth [2,3,4]. Such color alteration may occur after endodontic treatment and/or following trauma due to pulp hemorrhage and necrosis, or as a result of the use of certain endodontic sealing agents, drugs, or restorative materials [5,6,7]. Non-vital teeth appear to be darker and more saturated and have an increased hue interval, compared to their vital counterparts [1].

In these cases, internal bleaching is a conservative, simple, and effective treatment option, focused on lightening the color of the tooth with fast, safe and satisfactory results. Therefore, it is considered an ideal option to restore aesthetics and preserve the structural integrity of the tooth. In fact, it reduces the need for more invasive cosmetic procedures such as veneers and crowns [8].

This procedure involves the application of a chemical agent that oxidizes the organic pigmentation, eliminating stains by chromogenic degradation [9,10]. Larger chromogenic pigments break down into smaller, less intensive pigments, thus lightening the color of the teeth [11].

This treatment is applied using the “walking bleach” technique, a minimally invasive procedure that bleaches the devitalized, darkened tooth from the inside when bleaching agents are placed within the pulp chamber. Generally, it is done in several sessions. In the first, the bleaching product is placed inside the pulp chamber of the darkened devitalized tooth, and the cavity is sealed with a temporary restorative material, leaving it to act for about seven days. In each session, the bleaching product is changed and renewed (the one that has been allowed to act is removed and the same product is replaced) until the desired color is reached [12,13,14].

The most commonly used bleaching agents include carbamide peroxide (CP), hydrogen peroxide (HP), a mixture of sodium perborate (SP) with distilled water, or a combination of SP and HP, with HP being the active ingredient in all of them [15,16]. HP is the main oxidizing agent for tooth bleaching, a powerful biological oxidant for both organic and inorganic compounds, with the formation of reactive oxygen molecules, among others [5,13,14,15,16]. It can be applied directly on the tooth or produced locally in a chemical reaction from precursors such as SP or CP [13].

High concentrations of HP (≥30%) should only be used with caution, in order to avoid increasing the risk of root resorption during non-vital bleaching.

For that reason too, in recent years non-vital teeth have been bleaching with CP or SP. Bleaching agents tend to use higher concentrations for non-vital teeth than for in-office or at-home vital teeth bleaching.

Due to its low molecular weight, HP diffuses easily from the pulp chamber and can penetrate the dentine releasing oxygen which breaks the double bonds of organic and inorganic compounds within the dentinal tubules [11,17,18,19]. The deeper the penetration, the more pigment that causes the color alteration of dental tissues can be reversed by the oxidation reaction [20].

It has been proposed that the oxidative action of bleaching agents and releasing of nascent oxygen which can be transferred to the cervical periodontal ligament (PDL) through the dentinal tubules and cementum defects can act as a stimulus for inflammatory changes of the region and subsequent increase of odontoclastic cells responsible for invasive cervical root resorption [20].

On the other side, oxygen produced during internal bleaching may result in increased intracameral pressure due to the release of gas, causing loosening of temporary restorative materials placed between visits. Therefore, the marginal sealing of temporary restorations can be affected by bleaching reactions [13,21]

Loss of marginal integrity opens a microscopic space between the tooth and the restoration, leading to a cascade of events, such as leakage of bleaching components into the oral cavity, causing an acid taste, irritation, localized desquamation of the adjacent gingival and periodontal tissues, leakage of oral fluids and bacteria into the chamber cavity as well as a decrease in the effectiveness of the treatment [21,22,23,24,25,26]. The exact mechanism explaining peroxides action on each tooth substrate bleaching has not yet been fully elucidated [6,18,27,28], but over the years various authors have demonstrated that intracoronal bleaching agents also reach enamel [29,30].

To date, the expansion that occurs when the bleaching agent interacts with the dental tissue, especially critical when it comes to the temporary sealing of the cavity between visits, has not been quantified. The design of this study allows us to observe this expansive behavior in vitro, both in enamel and dentin, with different bleaching agents. The present study was carried out to evaluate, measure and compare the oxygen expansion that has when CP 37%, HP 30%, HP 30% + SP and SP interact with enamel and dentin, based on an experimental model, in order to better visualize and understand the release of oxygen that takes place and be able to make appropriate clinical decisions.

## 2. Materials and Methods

This experimental study was approved by the Ethics Committee of the Catholic University of Valencia (Valencia, Spain) (Ref.: UCV/2019-2020/037). This study is focused on the whitening of non-vital teeth, so we use higher concentrations of these products than would be used in vital teeth, in accordance with the European Directive 93/42/EEC.

The bleaching agents used are summarized in Table 1.

### 2.1. Sample Preparation

Thirty healthy human teeth free of cracks, caries, restorations and discolorations, and removed for orthodontic or periodontal reasons, were used in the study. Any traces of biofilm were removed by cleaning the teeth with gauze and a prophylaxis brush. The teeth were stored in phosphate buffered saline (PBS) solution (final concentration: 10 mM PO_4_^3^, 137 mM NaCl, 2.7 mM KCl) and were used within three months after extraction.

Either the enamel or the dentin component of each tooth was used as follows:

#### 2.1.1. Enamel Collection

The teeth were sectioned horizontally at the cemento-enamel junction (CEJ) to separate the crown from the root using a diamond disc drill (Hyperflex diamond disc, KOMET, Proclinic) at 300.000 rpm and cooled under water irrigation. A diamond conical drill (Diamond drill, model 856, Proclinic) was then used at high speed to eliminate the pulp tissue and dentin, leaving only the enamel component. Nikon SMZ-2T stereoscopic microscope (Nikon, Tokyo, Japan) and an Intralux 4000-1 light source (Volpi, Schlieren, Switzerland) were used to check that there were no remnants of dentin.

This group was included in the study, since the bleaching agents diffuse through the dentin tubules and can reach the enamel surface.

#### 2.1.2. Dentin Collection

A diamond conical drill was used to eliminate all of the enamel from the coronal surface, and following dentin exposure the teeth were sectioned horizontally in the same way as described above, to separate the crown from the root at the CEJ. The pulp tissue was then eliminated with a diamond conical drill, leaving only the coronal dentin. The same stereoscopic microscope was used to check that there were no remnants of enamel.

The different dental components (enamel and dentin) were placed separately in an electric mill (Nwouiiay 300 W—Electric Grinder, Guangdong, China) at 20,000 rpm, which was turned on intermittently to avoid heating, until a fine powder was obtained, with particles of an approximate size between 0.06 and 0.2 mm. This was done to be able to observe in vitro the expansive behavior that takes place after the interaction between dental tissues and bleaching agents.

**Figure 1:**
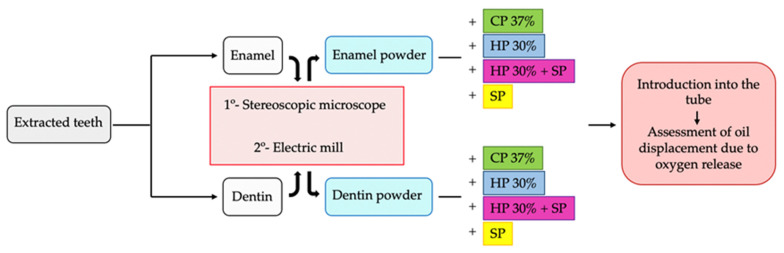
Flow chart with the summary of the main steps for the elaboration of the study groups, with enamel and dentin and the bleaching groups.

These steps are summarized in Figure 1 with the image of the flow chart.

Enamel and dentin were mixed with the four whitening groups, to assess whether there were significant differences in oxygen expansion between both dental tissues with the same bleaching agent, and to assess within the same tissue which agent produced greater oxygen expansion.

### 2.2. Experimental Design

**Figure 2:**
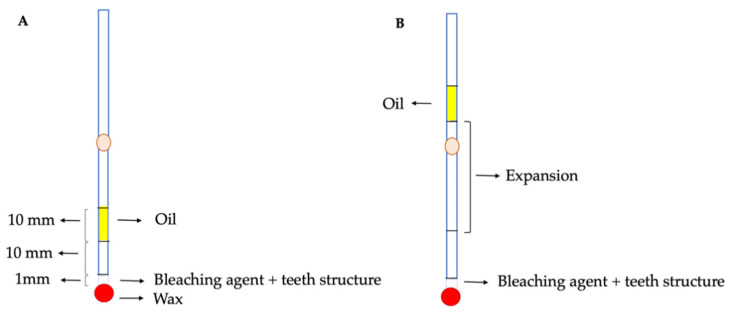
Experimental model at baseline, with the different components and oil in the starting position (**A**). Expansion in the tube, with displacement of the oil layer (**B**).

The experimental model used to investigate the expansive behavior of the internal bleaching agents consisted of glass tubes measuring 1 mm in diameter (2940211, Paul Marienfeld Superior, Alemania) (Figure 2). A syringe was used to inject a volume of oil occupying a length of 10 mm in the tube, with the purpose of containing the fluids. A volume of air occupying a length of 1 cm in the tube was interpositioned between the oil and the mixture of enamel or dentin with bleaching agents, in order to avoid any contact between the oil and the mixture. The bleaching material with dentin or enamel occupied a length of 1 mm along the tube, equivalent to a volume of 0.016 mL. The tube was then sealed with wax at the end of the tube opposite to where the oil was located. The expansion produced by the different materials was measured in millimeters, based on displacement of the oil within the tube.

The mixture was made with the help of a cement spatula and a dappen glass. The creamy consistency mixtures of enamel or dentin with the bleaching agents were distributed into the following groups, each composed of 30 tubes, also indicated in Table 1:

- Group 1: CP 37% (3 mL) with enamel (2 g).
- Group 2: CP 37% (3 mL) with dentin (2 g).
- Group 3: HP 30% (3 mL) with enamel (2 g).
- Group 4: HP 30% (3 mL) with dentin (2 g).
- Group 5: SP (1 g) mixed with HP 30% (3 mL) and enamel (1 g). (HP 30% + SP with enamel)
- Group 6: SP (1 g) mixed with HP 30% (3 mL) and dentin (1 g). (HP 30% + SP with dentin)
- Group 7: SP (1 g) and enamel (1 g) mixed with distilled water (3 mL).
- Group 8: SP (1 g) and dentin (1 g) mixed with distilled water (3 mL).

The amount of SP, enamel and dentin were calculated using a mini-scale (Accuweight-255EU).

A control of 30 tubes was established for each group, containing the bleaching agent but no enamel or dentin. The expansion produced by each of the bleaching agents as a consequence of gas release was evaluated every 24 h over 10 days, marking and measuring the displacement of the oil within the tube using a marker pen and a digital caliper. In the groups described, the bleaching agents are in accordance with the European Directive3/42/CEE.

### 2.3. Statistical Analysis

The statistical analysis of the data collected for the present study was carried out using the SPSS 23 computer program (IBM Corp, Armonk, NY, USA).

The results obtained from the oxygen expansion that occurs after the reaction between bleaching agent and tooth structure were subjected to statistical analysis.

On the one hand, expansion was measured and the data were analyzed to assess whether there were significant differences depending on the tooth structure for each bleaching agent. In the control groups of CP 37%, HP 30%, and SP, the oil did not move, and as no expansion was observed, we compared only each group of whitening with enamel and dentin using the Student’s t test.

In the control group, HP 30% + SP was the only control where there was oil displacement, so it was included in the statistical analysis to assess whether there were significant differences when interacting with enamel or dentin. To do this, we first used the robust test of equality of means (Welch’s ANOVA).

On the other hand, we compared within all groups of the same tooth structure (enamel or dentin) the different expansive reactions produced by each bleach using the Games-Howell post-hoc test.

## 3. Results

**Figure 3:**
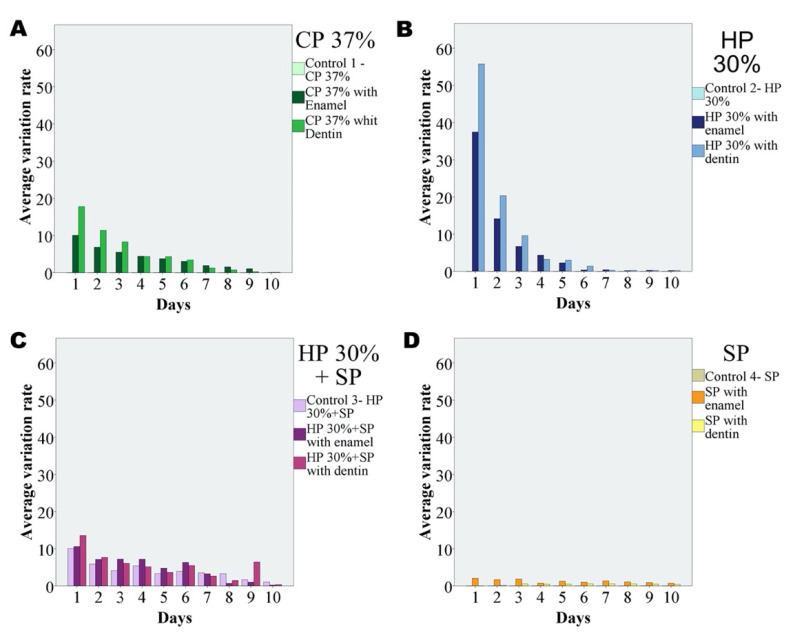
Time course of expansion over 10 days on mixing CP with enamel and dentin (**A**), HP with enamel and dentin (**B**), SP and HP with enamel and dentin (**C**), and SP with enamel and dentin (**D**).

A variation in volume with respect to the control was only observed in the group of HP 30% + SP. The results obtained in each of the groups are summarized in Figure 3 and Table 2.

### 3.1. Comparison of the Expansion between Enamel and Dentin for Each Bleaching Group

#### 3.1.1. CP 37%

On comparing the two groups with CP 37% as bleaching agent (Groups 1 and 2), the expansion produced was found to be significantly greater for CP with dentin than for CP with enamel (Student *t*-test; *p <* 0.05).

#### 3.1.2. HP 30%

On comparing the two groups with HP 30% as bleaching agent (Groups 3 and 4), the expansion produced was found to be significantly greater for HP with dentin than for HP with enamel (Student *t*-test; *p* < 0.05).

#### 3.1.3. HP 30% + SP

In this case the control group composed of SP and HP 30% was seen to produce expansion. No statistically significant differences were recorded among the three groups with HP 30% + SP as bleaching agents (Groups 5 and 6, and control) (ANOVA; *p* = 0.064).

**Figure 4:**
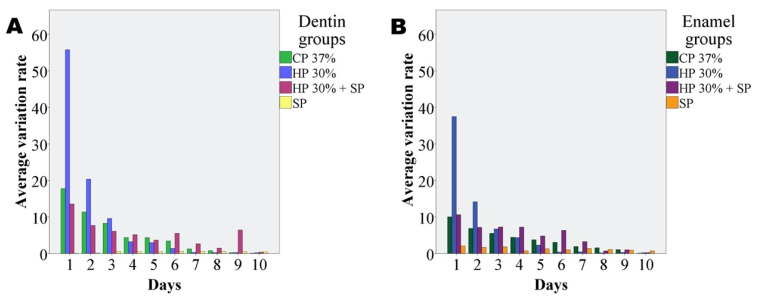
Time course per days of the expansion observed in mm through the tubes, resulting from the interaction between dentin (**A**) and enamel (**B**) with each bleaching group. An initial expansion is observed that decreases in the following days.

Although expansion was seen to increase on adding enamel or dentin, the difference over time failed to reach statistical significance. Likewise, the expansion observed with the mixture containing dentin was not significantly different from that recorded with the mixture containing enamel. Figure 4 shows the variation of expansion over the 10 days of the study corresponding to the different bleaching agents with enamel and dentin.

#### 3.1.4. SP

In general, expansion in the groups of SP with enamel and dentin (Groups 7 and 8) proved homogeneous over time. In this case, the expansion produced was found to be significantly greater for SP with enamel than for SP with dentin (Student t-test; *p <* 0.05).

The results obtained in each of the groups with enamel and dentin are summarized in Table 3.

### 3.2. Comparison of Expansion of the Different Bleaching Agents with Enamel

The Games-Howell post hoc test for the comparison of means showed expansion in the SP with enamel group to be significantly smaller than in the rest of the groups of bleaching agents with enamel, while that recorded in the HP 30% with enamel group was significantly greater (Table 4).

### 3.3. Comparison of Expansion of the Different Bleaching Agents with Dentin

The Games-Howell post hoc test for the comparison of means showed expansion in the SP with dentin group to be significantly smaller than in the rest of the groups of bleaching agents with dentin, while that recorded in the HP 30% with dentin group was significantly greater (Table 5).

The results obtained in each of the groups are summarized in Table 4 and Table 5.

The amount of expansion was decreasing over time, for all of the whitening groups, showing a decreasing trend with the days.

## 4. Discussion

The present study examined the expansive behavior resulting from the interaction of different bleaching agents with enamel and dentin.

The use of the stereomicroscope to analyze dental tissues and assess the extent of their lesions (caries, wear, leakage of restorations…) is described in the literature [31,32,33], so we used this method to check the sample was entirely of enamel or dentin, before grinding it. There are studies in the literature in which this procedure is also performed to compare the behavior of a certain product on each structure [16,28].

In order to be able to observe and measure in vitro the release of oxygen that takes place after the interaction of the bleaching agent and the tooth structure, the enamel and dentin were crushed separately to obtain a fine powder that was introduced into the tube for in vitro study.

Controls composed only of the bleaching agent without any tooth structure did not produce expansion, since the bleaching agent alone does not produce any reaction, and this occurs and is observed when they interact with a tooth structure.

In the control group HP 30% + SP, there was an oil displacement, a fact that means that both bleaching agents together interact, releasing oxygen from the beginning. This fact is described in the literature [34,35].

In 1961, Spasser recommended the use of SP within the pulp chamber [36]. In 1967, his technique was modified by Nutting and Poe, who substituted HP 30% for water and proposed that the term “walking bleach” be used to refer to the technique [35]. They suggested this change precisely because the mix between these two agents released oxygen, and they thought it might be more effective for internal bleaching.

In this study, we observed that this interaction occurs when both agents are mixed, and that the oxygen release observed is not significantly different when including enamel or dentin. This fact could be taken into account for further studies.

All of the bleaching agents evaluated produced expansion to one degree or another when interacting with dental tissues. CP 37% and HP 30% showed greater significant expansion in dentin than in enamel. This could be due to the difference in organic content between the two structures, according to a study by Eimar et al. in which they studied the bleaching mechanism on enamel [28]. The results of their study showed that HP whitens teeth by oxidizing the organic matrix of enamel. In addition, they indicated that it was expected that HP could have a similar or even stronger effect on dentin than what they observed on enamel, due to the higher amount of organic content. In this study we agree with their indications, showing that the expansive reaction that occurs when oxygen is released is greater in dentin than in enamel. Therefore, the contrasted variability in tooth organic content among people [37] could be one of the reasons behind the wide variation in results obtained following tooth bleaching treatment. More studies are needed to elucidate the behavior of the combination of HP 30% + SP and SP.

The oxygen and free radicals establish their primary mechanism of action in tooth bleaching [18,38,39,40]. The beginning of the expansive process could be observed almost immediately, from the first hour. However, it was gaining strength and was more evident in all groups within the first 24 h.

Then it gradually decreased with the exception of the SP group, where the expansion was relatively homogeneous over time and of lesser magnitude than in the other groups.

The marginal sealing of temporary restorations can be affected by bleaching reactions. The gas released after the bleaching reaction can increase the pressure within the pulp chamber, increasing the possibility of loosening or displacing the temporary restoration, opening a microscopic space between the restoration and the tooth. This could cause, among other things already mentioned, bacterial leakage, and endodontic treatment failure, as well as a prolongation of treatment with more sessions due to not obtaining the desired results [13,21,25,41].

Another aspect is the importance of the coronal seal so that the expansion of oxygen facilitates the penetration of the bleaching agent into the dentin tubules and it is not lost due to a poor coronal sealing [7]. Some studies underscored the relevance of good coronal sealing with enamel etching, adhesive and composite [42,43,44], so that the force with which the oxygen is released does not misalign the provisional restoration, expel it into the oral cavity and whitening results ineffective. In addition, a good seal prevents recontamination of the dentin with microorganisms and reduces the risk of renewed staining. It has also been suggested that accidental ingestion of HP at high concentrations may cause adverse gastric reactions [45].

Hosoya et al. comparing in vitro the sealing capacity of five materials used as a temporary sealing agent for the walking bleach technique demonstrated that coronal sealing is a critical procedure during the walking bleach technique [21,38].

The concern of bacterial leakage due to the mismatch of the provisional material has been a studied topic, and there are articles focused on using agents such as chlorhexidine together with bleaching products to act against these microorganisms, but the expansion that occurs when interacting whitening agents with dental tissues has not been quantified [21].

Although different theories have been proposed to explain the appearance of invasive cervical resorption (ICR) after treatment, [39] it seems clear that the bleaching agent first must reach the periodontal ligament [40]. Diffusion has been cited as the underlying mechanism, understood as the displacement of a substance from one space to another down a concentration gradient, to explain HP transfer to the external surface of the tooth in the context of intracoronal bleaching [42,46,47]. However, we have found no mention in the literature of the expansion produced by the force of interaction of bleaching agents with enamel and dentin as a possible additional cause of bleaching agent transfer to the exterior of the tooth. Such expansive action could prove important in this respect.

The results of this study coincide with the diffusion observed in other studies [47,48,49]. Rokaya et al. used spectrophotometry with potassium permanganate to analyze the extra-radicular diffusion of HP associated to intracoronal bleaching. After 24 h, the group exposed to HP 35% yielded significantly greater extra-radicular diffusion, while the SP group showed diffusion of significantly lesser magnitude. These results coincide with our own observations in these same two groups; expansion therefore could contribute to explain HP displacement to the exterior of the tooth. Other authors have also found peak HP diffusion to occur after 24 h in coincidence with our own observations [48].

Gokay et al. reported the level of penetration of CP to be significantly lesser than that of the combination HP 30% + SP [42]. In our study we observed similar results in terms of oxygen expansion in the case of enamel. However, the cited studies have different methodologies compared to the present study, so the results should be compared with caution. The possible association could be assessed in future studies.

On the other hand, Lee et al. reported greater diffusion with SP than with CP [50].

Camps et al. recorded the diffusion over time of HP through the dentinal layer of the teeth of young or adult patients using a CP 20% gel, in order to determine the optimum renewal rate [51]. Their results suggested that HP exhibits a greater diffusion rate over time in younger individuals, since their dentinal tubules are of greater diameter. These data, together with our observations regarding the oxygen release observed when enamel and dentin interact with bleaching agents, suggest that SP may be the bleaching agent of choice for young patients with wider dentinal tubules, though the adverse effects of internal bleaching consequently could also be greater in such individuals.

In general, better results appear to be obtained in internal whitening when the SP is mixed with another bleaching agent such as HP or CP. De Souza-Zaroni et al. compared the bleaching efficacy of SP mixed with CP 37% and SP for intracoronal bleaching, observing better results with the mixture of both bleaches combined than with SP alone. [4]

Lim y cols. compared the whitening efficacy of CP 35%, HP 35%, and SP for intracoronal whitening of discolored root-filled teeth. CP 35% and HP 35% were equally effective for intracoronal bleaching, and significantly better than SP after seven days [52].

These results on the bleaching efficacy are consistent with this in vitro study. HP 30% shows significantly greater oxygen expansion than the other agents for both enamel and dentin, and SP shows significantly less expansion for both as well. The expansion in the group CP 37% with dentin was not significantly different from that observed with HP 30% + SP. However, with the enamel group, a significantly greater expansion was observed using HP 30% + SP than with CP 37%. It would be interesting to observe in future studies why SP seems to interact more with enamel.

Carrasco et al. evaluated the effect of CP 37%, CP 27%, and HP 20% mixed with SP on the permeability of the dentin of non-vital anterior teeth [20]. Apparently, it was observed that with higher concentration, better dentin permeability resulted. The greatest increase in dentin permeability was produced by CP 37%, followed by HP 20% with SP. CP 27% produced the lowest results of these groups in the alteration of dentinal permeability. These results could also be extrapolated to those of our study. The higher the concentration of bleach, the greater the expansion of oxygen observed.

This in vitro study allows us to observe, quantify and compare the expansive behavior that occurs when CP 37%, HP30%, HP30% + SP, and SP interact with enamel and dentin separately. These results highlight the marked oxygen expansion reaction that takes place and improve the understanding of why we can find certain aspects described in the literature: The unwanted effect of displacement or loosening of the temporary restoration, with the specific repercussions indicated that this may have (localized irritation and desquamation of gingival and periodontal tissues, failure of whitening treatment, leakage of bacteria and failure of endodontic treatment…) also a possible explanation of how the agent penetrates the dentin tubules and enamel and the influence of its organic component. More future research is needed to verify and link the concepts that have been observed.

## 5. Conclusions

The bleaching action is attributed to the oxidation reaction between the bleaching agent and the tooth structure (enamel or dentin) measured in this study thanks to the observed expansion in the tubular model. We found different clinical implications derived from the results of this study. Since there is a greater expansion reaction with dentin than with enamel, it is reasonable to think that it is due to the fact that HP interacts with the organic component. This expansive reaction can dislodge the temporary restoration, causing bacterial leakage and contamination of the pulp chamber and root canal, and the failure or decreased effectiveness of the whitening treatment, so it is important to try to prevent these complications. Furthermore, it is also observed that the strength of the reaction is related to the concentration of the bleaching agent.

## Figures and Tables

**Table 1:** Summary table with each of the bleaching groups, abbreviations, and information on bleaching agents.

(A) Bleaching Agents	Abbreviation	Information
Carbamide peroxide (37%) (CH_4_N_2_O·H_2_O_2_) gel	CP 37%	Whiteness Super 37 FGM, Joinville, SC, Brazil
Hydrogen peroxide (30%) (H_2_O_2_) liquid	HP 30%	Foret, Peroxfarma, Spain
Sodium perborate (NaBO_3_) powder	SP	Acofarma Distribution, Spain
**(B) Bleaching groups**		
CP 37%	Control: CP 37% (*n* = 30)	
	CP 37% with enamel powder (*n* = 30)	
	CP 37% with dentin (*n* = 30)	
HP 30%	Control: HP 30% (*n* = 30)	
	HP 30% with enamel powder (*n* = 30)	
	HP 30% with dentin powder (*n* = 30)	
HP 30% + SP	Control: HP 30% + SP (*n* = 30)	
	HP 30% + SP with enamel powder (*n* = 30)	
	HP 30% + SP with dentin powder (*n* = 30)	
SP	Control: SP with distilled water (*n* = 30)	
	SP with distilled water and enamel powder (*n* = 30)	
	SP with distilled water and dentin powder (*n* = 30)	

**Table 2:** Summary data of the total sample after 10 days, expressed in the distance (mm) that the oil rose in the tube due to the expansion caused by the mixture.

Enamel			
Bleaching Agents	Mean	Min.	Max.
CP 37% (*n* = 30)	38.4	15	61
HP 30% (*n* = 30)	66.6	30	104
HP 30% + SP (*n* = 30)	48.6	38	67
SP (*n* = 30)	12.7	7	24
**Dentin**			
CP 37% (*n* = 30)	52.06	35	69
HP 30% (*n* = 30)	94.5	58	158
HP 30% + SP (*n* = 30)	52.7	21	122
SP (*n* = 30)	4.4	2	7
**Control**			
CP 37% (*n* = 30)	0	0	0
HP 30% (*n* = 30)	0	0	0
HP 30% + SP (*n* = 30)	42,3	19	58
SP (*n* = 30)	0	0	0

**Table 3:** Comparison of the results obtained between the different groups with enamel and dentin, regarding the elevation of the oil inside the tube produced by the expansion of each mixture.

Enamel			Dentin		
Group (*n* = 30)	Comparative (*n* = 30)	*p*-Value	Group (*n* = 30)	Comparative (*n* = 30)	*p*-Value
CP 37%	HP 30%	<0.001	CP 37%	HP 30%	<0.001
	HP 30% + SP	0.004		HP 30% + SP	1.000
	SP	<0.001		SP	<0.001
HP 30%	CP 37%	<0.001	HP 30%	CP 37%	<0.001
	HP 30% + SP	<0.001		HP 30% + SP	<0.001
	SP	<0.001		SP	<0.001
HP 30% + SP	CP 37%	0.004	HP 30% + SP	CP 37%	1.000
	HP 30%	<0.001		HP 30%	<0.001
	SP	<0.001		SP	<0.001
SP	CP 37%	<0.001	SP	CP 37%	<0.001
	HP 30%	<0.001		HP 30%	<0.001
	HP 30% + SP	<0.001		HP 30% + SP	<0.001

**Table 4:** Multiple comparisons of enamel bleaching groups. Multiple comparisons of enamel bleaching groups. Summary of the statistical analysis between each of the enamel bleaching groups, with the Games-Howell contrast test after 10 days.

Multiple Comparisons of Enamel Bleaching Groups						
Groups with Enamel (E_1_)	Enamel Comparison Groups (E_2_)	Difference of Means (E_1_–E_2_)	Typical Error	*p*-Value	95% Confidence Interval	
					Lower Limit	Upper Limit
CP 37%	HP 30%	28.294 *	4.056	0.000	−39.04	−17.55
	HP 30% + SP	−10.255 *	2.831	0.004	−17.80	−2.71
	SP	25.604 *	2.634	0.000	18.52	32.69
HP 30%	CP 37%	28.294 *	4.056	0.000	17.55	39.04
	HP 30% + SP	18.039 *	3.498	0.000	8.66	27.42
	SP	53.898 *	3.341	0.000	44.87	62.93
HP 30% + SP	CP 37%	10.255 *	2.831	0.004	2.71	17.80
	HP 30%	−18.039 *	3.498	0.000	−27.42	−8.66
	SP	35.859 *	1.652	0.000	31.47	40.25
SP	CP 37%	−25.604 *	2.634	0.000	−32.69	−18.52
	HP 30%	−53.898 *	3.341	0.000	−62.93	−44.87
	HP 30% + SP	−35.859 *	1.652	0.000	−40.25	−31.47

Data marked with (*) indicate statistical significance: (*p*-value < 0.005).

**Table 5:** Multiple comparisons of dentin bleaching groups. Summary of the statistical analysis between each of the dentin bleaching groups, with the Games-Howell contrast test after 10 days.

Multiple Comparisons of Dentin Bleaching Groups						
Groups with Dentin (D_1_)	Dentin Comparison Groups (D_2_)	Difference of Means (D_1_–D_2_)	Typical Error	*p*-value	95% Confidence Interval	
					Lower Limit	Upper Limit
CP 37%	HP 30%	−42.455 *	5.325	0.000	−56.80	−28.11
	HP 30% + SP	**−0.640**	6.239	1.000	−17.50	16.21
	SP	47.617 *	1.753	0.000	42.85	52.38
HP 30%	CP 37%	42.455 *	5.325	0.000	28.11	56.80
	HP 30% + SP	41.814 *	7.827	0.000	21.09	62.54
	SP	90.072 *	5.041	0.000	76.34	103.80
HP 30% + SP	CP 37%	**0.640**	6.239	1.000	−16.21	17.50
	HP 30%	−90.072 *	5.041	0.000	−103.80	−76.34
	SP	48.257 *	5.998	0.000	31.92	64.60
SP	CP 37%	−47.617 *	1.753	0.000	−52.38	−42.85
	HP 30%	**−90.072**	5.041	0.000	−103.80	−76.34
	HP 30% + SP	−48.257 *	5.998	0.000	−64.60	−31.92

Data marked with (*) indicate statistical significance: (*p*-value < 0.005).

## Data Availability

Data is available upon reasonable request.
